# Bronchopneumonia With Cutaneous Vasculitis: A Diagnostic Challenge

**DOI:** 10.7759/cureus.114730

**Published:** 2026-08-18

**Authors:** Aayush Goel, Aneet Mahendra, Sameer Singhal, Sanjeev Gupta, Vishesh Dhawan, Jhanvi Goyal

**Affiliations:** 1 Department of Dermatology, Maharishi Markandeshwar Institute of Medical Sciences and Research, Maharishi Markandeshwar (Deemed to Be University), Ambala, IND; 2 Department of Respiratory Medicine, Maharishi Markandeshwar Institute of Medical Sciences and Research, Maharishi Markandeshwar (Deemed to Be University), Ambala, IND; 3 Department of Pathology, Maharishi Markandeshwar Institute of Medical Sciences and Research, Maharishi Markandeshwar (Deemed to Be University), Ambala, IND

**Keywords:** bronchopneumonia, clinicopathological correlation, cutaneous vasculitis, erythema nodosum, leukocytoclastic vasculitis, multidisciplinary approach

## Abstract

Bronchopneumonia associated with atypical cutaneous manifestations poses a significant diagnostic challenge because of the broad spectrum of infectious, inflammatory, autoimmune, and vasculitic disorders that may present with overlapping clinical features. Careful clinicopathological correlation is often required to establish the diagnosis and identify the underlying etiology.

A 73-year-old man presented with fever, productive cough, progressive dyspnea, and bilateral pulmonary infiltrates. During hospitalization, he developed painful erythematous plaques over the left leg accompanied by hemorrhagic lesions over both palms and the left sole. The initial clinical differential diagnosis included erythema nodosum and urticarial vasculitis. An extensive evaluation, including bronchoscopy with microbiological investigations, autoimmune serology, and blood cultures, did not identify a definitive underlying cause. Skin biopsy demonstrated features suggestive of cutaneous vasculitis, establishing the diagnosis following clinicopathological correlation. The patient showed progressive improvement with systemic corticosteroid therapy, with parallel resolution of both the cutaneous lesions and pulmonary abnormalities.

This case highlights the importance of a systematic multidisciplinary approach in patients presenting with concurrent bronchopneumonia and atypical cutaneous manifestations. Integrating clinical, radiological, microbiological, serological, and histopathological findings is essential for distinguishing among infectious, inflammatory, autoimmune, and vasculitic disorders and for guiding appropriate management.

## Introduction

Bronchopneumonia may be associated with a wide spectrum of cutaneous manifestations, often posing a diagnostic challenge because several infectious, inflammatory, autoimmune, drug-induced, and neoplastic conditions can involve both the lungs and the skin [1]. Painful erythematous plaques over the leg accompanied by hemorrhagic palmoplantar lesions are an uncommon clinical presentation and considerably broaden the differential diagnosis. Establishing the correct diagnosis in such patients requires careful integration of the clinical presentation with laboratory, radiological, and histopathological findings.

Erythema nodosum (EN) is a common cause of painful erythematous lesions over the legs and is classically characterized histologically by septal panniculitis without vasculitis [2]. However, cutaneous vasculitis may occasionally present with a similar clinical appearance, making distinction between these entities difficult on clinical grounds alone. Skin biopsy, interpreted in conjunction with the clinical findings, plays a pivotal role in establishing the diagnosis when the clinical presentation is atypical and guiding further evaluation. We report an elderly patient with pneumonia-like illness who developed EN-like plaques accompanied by hemorrhagic palmoplantar lesions, illustrating the diagnostic challenges posed by this unusual clinical presentation.

## Case presentation

A 73-year-old man presented to the Department of Respiratory Medicine with a two-month history of intermittent high-grade fever with chills, shortness of breath, and productive cough for eight days. He denied hemoptysis, chest pain, recent drug intake, contact with tuberculosis, or prior antitubercular therapy. He had a history of COVID-19 infection in 2021 and was a chronic smoker with a 35-pack-year smoking history. Initial evaluation revealed bilateral pulmonary infiltrates on chest radiography.

Approximately two weeks before admission, the patient noticed a solitary painful erythematous lesion over the medial aspect of the left leg, followed by the appearance of additional similar lesions over the same limb. During hospitalization, he developed painful hemorrhagic plaques over both palms and the left sole, following which a dermatology consultation was sought. Cutaneous examination revealed multiple erythematous to violaceous, tender, indurated plaques over the lower limb (Figure 1A). In addition, well-defined hemorrhagic plaques with central dusky discoloration were present over both palms and the left sole (Figure 1B). Mucosal examination was unremarkable, and there was no peripheral lymphadenopathy.

**Figure 1:**
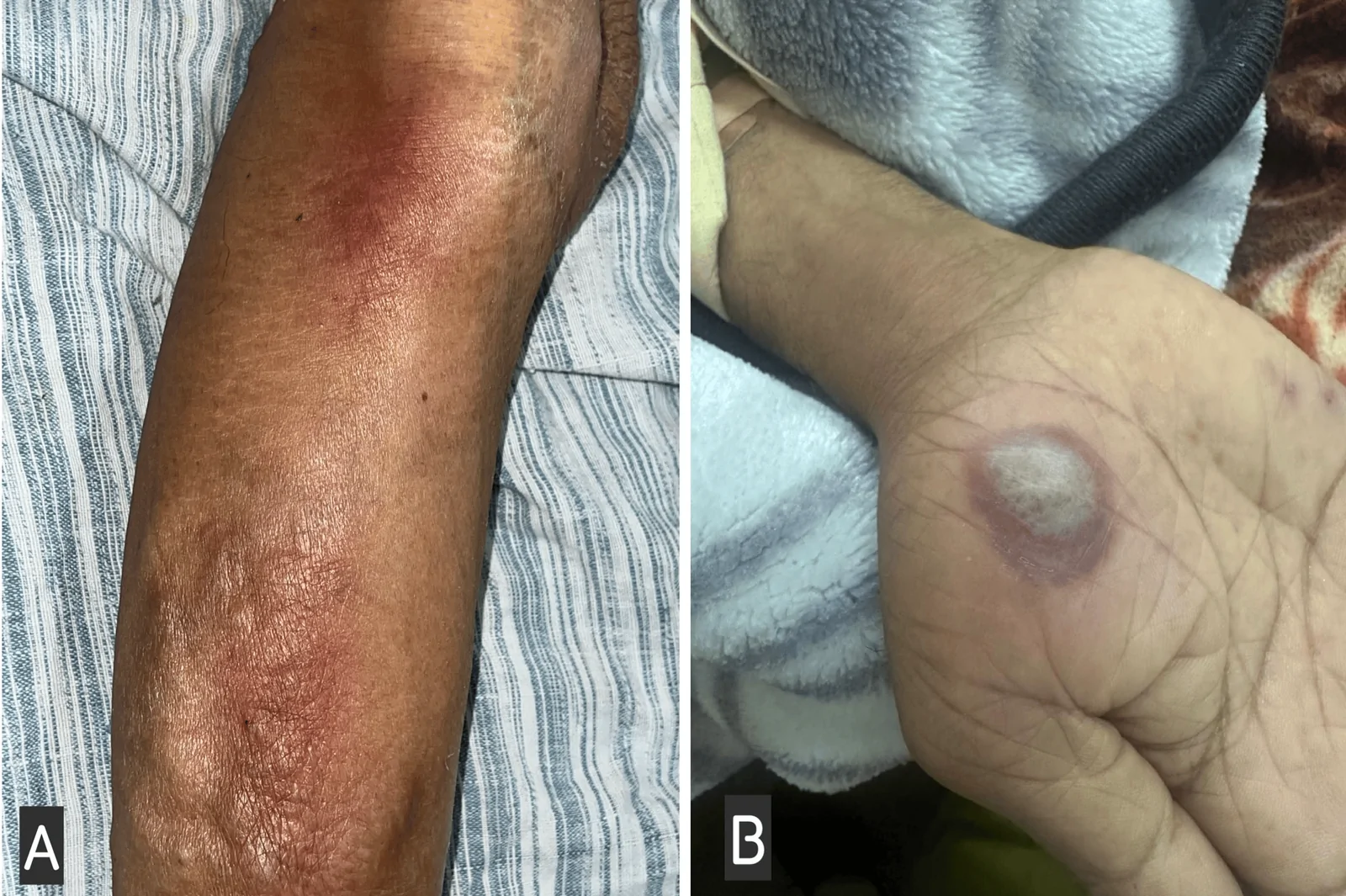
Clinical presentation of the cutaneous lesions (A) Erythematous plaques over the left lower limb. (B) Hemorrhagic plaque with central dusky discoloration over the left palm

Given the coexistence of pulmonary and cutaneous manifestations, a comprehensive evaluation was undertaken to identify a common underlying etiology. High-resolution computed tomography of the chest demonstrated bilateral inflammatory parenchymal changes superimposed on chronic fibrotic lung changes, consistent with an active infective or inflammatory process. Laboratory investigations demonstrated normocytic normochromic anemia, reactive thrombocytosis, elevated C-reactive protein, and fluctuating leukocyte counts during hospitalization, consistent with an active systemic inflammatory response. Serological tests for HIV, hepatitis B, and hepatitis C were nonreactive.

Microbiological evaluation, including sputum smear examination for acid-fast bacilli (AFB), gastric aspirate for AFB, Ziehl-Neelsen staining, and cartridge-based nucleic acid amplification test (CBNAAT), was negative for *Mycobacterium tuberculosis*. Bronchoscopy with bronchoalveolar lavage (BAL) demonstrated only reactive bronchial epithelial cells without evidence of malignancy. BAL samples were negative for AFB, while bacterial and fungal cultures did not reveal a significant respiratory pathogen. Blood cultures remained sterile. Autoimmune evaluation, including antinuclear antibody, rheumatoid factor, anticyclic citrullinated peptide antibody, perinuclear antineutrophil cytoplasmic antibody, and cytoplasmic antineutrophil cytoplasmic antibody, was negative. Collectively, these findings excluded tuberculosis and common autoimmune causes of panniculitis and vasculitis. In the absence of a definitive microbiological diagnosis, bronchopneumonia, possibly of viral origin, was considered. The key laboratory and ancillary investigations are summarized in Tables 1, 2.

**Table 1:** Summary of quantitative laboratory investigations

Category	Investigation	Result	Reference range
Inflammatory markers	Erythrocyte sedimentation rate	40 mm/hour	<20 mm/hour
	C-reactive protein	56.3 mg/L	<5 mg/L
Autoimmune evaluation	Rheumatoid factor	2.7 IU/mL (negative)	<20 IU/mL
	Anticyclic citrullinated peptide antibody	7 U/mL (negative)	<17 U/mL

**Table 2:** Summary of qualitative laboratory and ancillary investigations AFB: acid-fast bacilli; CBNAAT: cartridge-based nucleic acid amplification test; HbsAg: hepatitis B surface antigen; HCV: hepatitis C virus; ANA: antinuclear antibody; p-ANCA: perinuclear antineutrophil cytoplasmic antibody; c-ANCA: cytoplasmic antineutrophil cytoplasmic antibody

Category	Investigation	Result
Microbiological evaluation	Blood culture	Sterile
	Sputum smear for AFB	Negative
	Gastric aspirate smear for AFB	Negative
	Gastric aspirate CBNAAT	Negative
Bronchoalveolar lavage	Smear for AFB	Negative
	CBNAAT	Negative
	Bacterial culture	No significant pathogen isolated
	Fungal culture	No significant fungal growth
	Cytology	Negative for malignant cells
Viral serology	HIV	Nonreactive
	HBsAg	Nonreactive
	HCV	Nonreactive
Autoimmune evaluation	ANA	Negative
	p-ANCA	Negative
	c-ANCA	Negative

Skin biopsy from the lower limb demonstrated inflammatory infiltrates involving and surrounding dermal blood vessels, with fibrinoid necrosis and erythrocyte extravasation, consistent with cutaneous vasculitis. Biopsy from the palmar lesion revealed full-thickness epidermal necrosis with features compatible with leukocytoclastic vasculitis. These findings, interpreted in conjunction with the clinical presentation, established the diagnosis. Representative histopathological features are shown in Figure 2.

**Figure 2:**
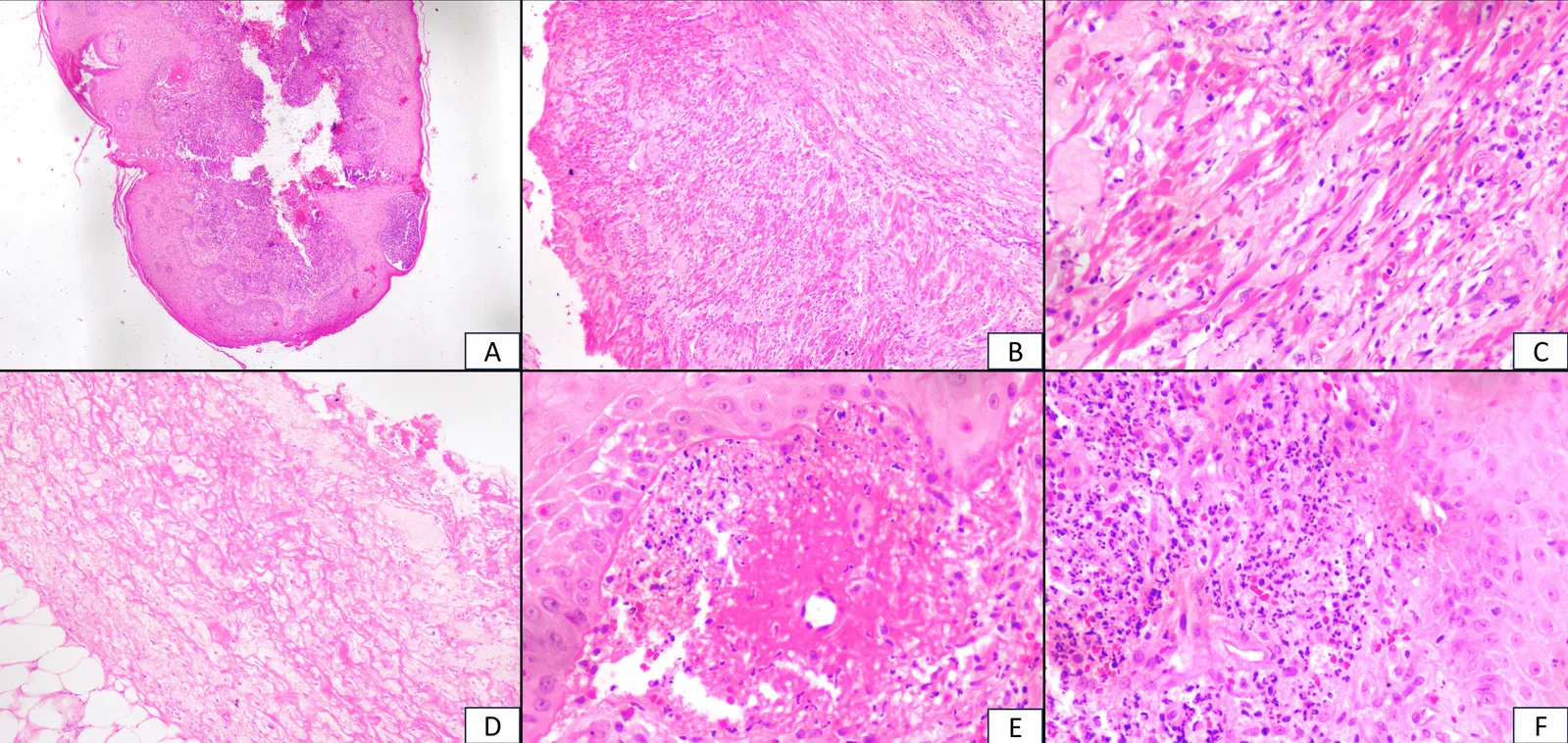
Histopathological findings from the skin biopsies (A) Full-thickness epidermal necrosis involving the upper dermis (H&E, ×40). (B,C) Presence of inflammatory cells within and around vessel wall (H&E, ×100 and ×400, respectively). (D) Homogenous eosinophilic changes in the subcutis (H&E, ×100). (E) Fibrinoid necrosis of the vessel wall (H&E, ×400). (F) Erythrocyte extravasation (H&E, ×400) H&E: hematoxylin and eosin

Following multidisciplinary management with systemic corticosteroids, antimicrobial therapy, and supportive care, the patient demonstrated progressive clinical improvement. Constitutional and respiratory symptoms resolved, accompanied by gradual healing of the leg lesions and hemorrhagic palmoplantar lesions. Serial chest radiographs confirmed resolution of the pulmonary infiltrates, mirroring the improvement in the cutaneous findings (Figure 3). He was discharged on a tapering regimen of oral methylprednisolone and remained clinically stable on follow-up, with no recurrence of skin lesions or respiratory symptoms.

**Figure 3:**
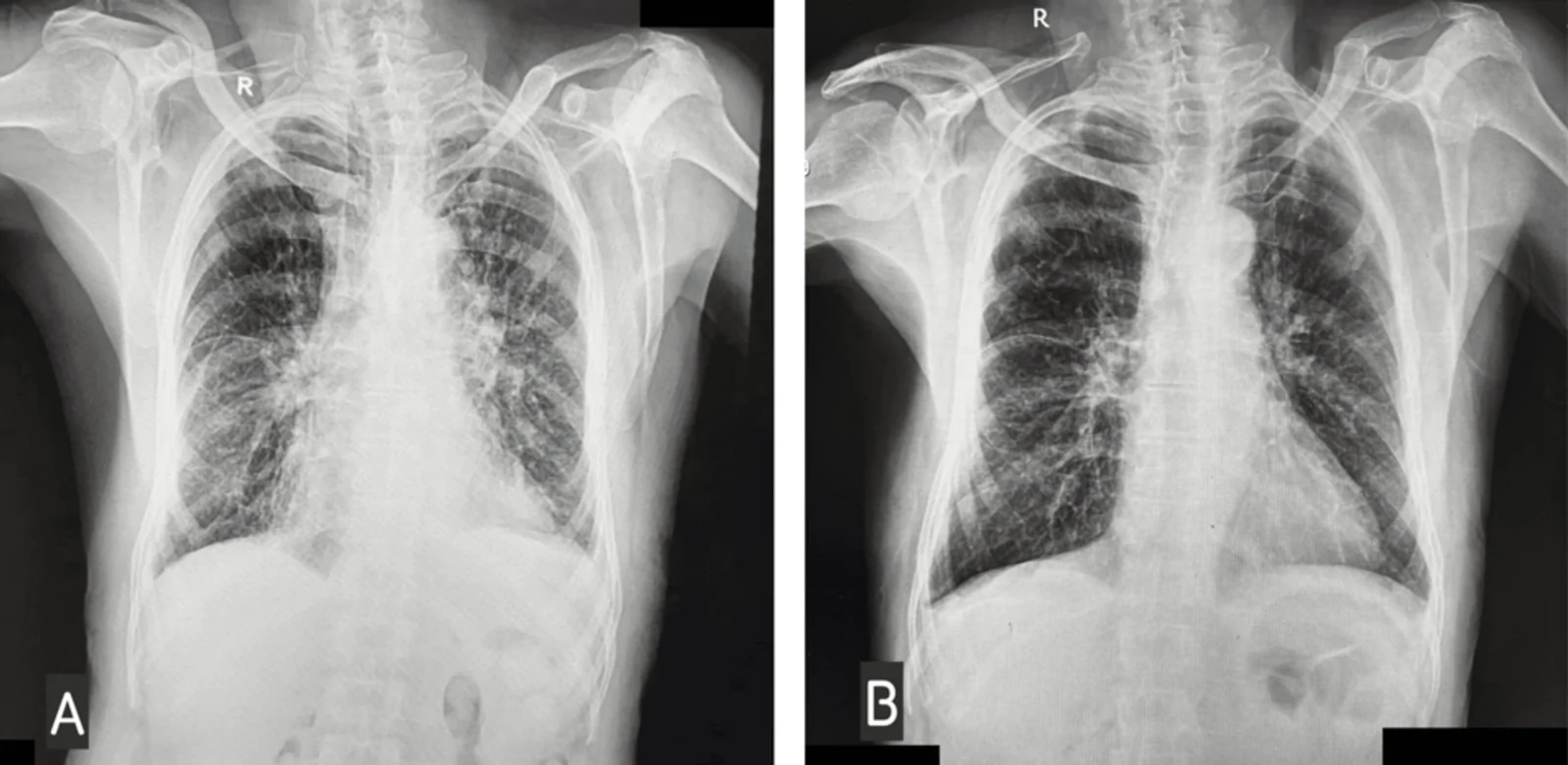
Chest radiographs demonstrating interval improvement in pulmonary infiltrates (A) Baseline posteroanterior chest radiograph demonstrating bilateral interstitial pulmonary infiltrates, more prominent in the left perihilar and bilateral lower lung fields. (B) Follow-up posteroanterior chest radiograph showing marked resolution of the bilateral pulmonary infiltrates, with minimal residual interstitial prominence

## Discussion

The simultaneous occurrence of bronchopneumonia and skin lesions presents a broad diagnostic spectrum, requiring exclusion of infectious, autoimmune, inflammatory, and neoplastic disorders [1,3]. In our patient, the presence of pulmonary infiltrates with constitutional symptoms initially raised the possibility of an underlying infectious process, particularly tuberculosis, while the accompanying cutaneous findings prompted consideration of systemic vasculitis and connective tissue diseases [4,5]. The presence of fever, productive cough, bilateral pulmonary infiltrates, and elevated inflammatory markers supported the diagnosis of bronchopneumonia. Despite extensive microbiological evaluation, bronchoscopy, autoimmune serology, and blood cultures, no definitive etiology could be established.

The temporal association between the pulmonary illness and the onset of cutaneous lesions, together with the parallel clinical and radiological improvement following multidisciplinary management, suggested a common immune-mediated inflammatory process [6]. Although microbiological confirmation of an infectious trigger was lacking, infections remain well-recognized precipitants of secondary cutaneous vasculitis, presumably through immune complex-mediated vascular injury [7]. In the present case, a respiratory infection was considered the most plausible trigger after exclusion of other common causes.

The dermatological presentation further added to the diagnostic complexity. While the painful erythematous plaques over the leg were clinically suggestive of EN, the concurrent hemorrhagic palmoplantar lesions were atypical and prompted skin biopsy. Skin biopsy findings were consistent with cutaneous vasculitis and, when interpreted in the context of the clinical presentation, redirected the diagnostic consideration [8]. This emphasizes that clinical examination alone may be insufficient in patients with concurrent systemic illness.

This case illustrates the diagnostic complexity of bronchopneumonia associated with atypical cutaneous manifestations [1]. Although the initial clinical impression favored EN because of the painful erythematous lesions over the leg, the concurrent hemorrhagic palmoplantar lesions together with biopsy findings consistent with cutaneous vasculitis prompted reconsideration of the diagnosis [8]. It also highlights that, when extensive investigations fail to identify a definitive underlying etiology, multidisciplinary assessment and close clinicopathological correlation are essential for arriving at the correct diagnosis and guiding appropriate management.

## Conclusions

Patients presenting with bronchopneumonia and atypical cutaneous manifestations should be evaluated using a structured, multidisciplinary approach that integrates clinical, radiological, microbiological, serological, and histopathological findings. In our patient, this comprehensive evaluation established cutaneous vasculitis despite an initial clinical impression of EN, illustrating the importance of clinicopathological correlation. Such an approach is essential for accurately distinguishing among infectious, inflammatory, autoimmune, and vasculitic disorders and for guiding appropriate management.
